# Nanomagnetic Gene Transfection for Non-Viral Gene Delivery in NIH 3T3 Mouse Embryonic Fibroblasts

**DOI:** 10.3390/ma6010255

**Published:** 2013-01-18

**Authors:** Angeliki Fouriki, Jon Dobson

**Affiliations:** 1 Institute for Science & Technology in Medicine, Keele University, Thornburrow Drive, Hartshill, Stoke-on-Trent ST4 7QB, UK; E-Mail: a.fouriki@keele.ac.uk; 2 J. Crayton Pruitt Family Department of Biomedical Engineering and Department of Materials Science & Engineering, University of Florida, P.O. Box 116400, Gainesville, FL 32611, USA; 3 Institute for Cell Engineering and Regenerative Medicine (ICERM), University of Florida, P.O. Box 116131, Gainesville, FL 32611, USA

**Keywords:** non-viral gene transfection, nanomagnetic gene transfection, DNA, magnetic nanoparticles, magnetic field

## Abstract

The objective of this work was to examine the potential of oscillating nanomagnetic gene transfection systems (magnefect-nano™) for improving the transfection efficiency of NIH3T3 mouse embryonic fibroblasts (MEFs) in comparison to other non-viral transfection techniques—static magnetofection™ and the cationic lipid agent, Lipofectamine 2000™. Magnetic nanoparticles (MNPs) associated with the plasmid coding for green fluorescent protein (GFP) were used to transfect NIH3T3 cells. The magnefect-nano system was evaluated for transfection efficiency, and any potential associated effects on cell viability were investigated. MNPs associated with the plasmid coding for GFP were efficiently delivered into NIH3T3 cells, and the magnefect-nano system significantly enhanced overall transfection efficiency in comparison to lipid-mediated gene delivery. MNP dosage used in this work was not found to affect the cell viability and/or morphology of the cells. Non-viral transfection using MNPs and the magnefect-nano system can be used to transfect NIH3T3 cells and direct reporter gene delivery, highlighting the wide potential of nanomagnetic gene transfection in gene therapy.

## 1. Introduction

In recent years, conventional therapies have provided limited improvement in treating complex diseases, creating an increasing need for the investigation of gene therapy as a potential tool for clinical and/or scientific research purposes [1,2,3].

Despite the success of viral vectors at transfecting a variety of primary cells and cell lines, over the past decade, there has been an increasing shift towards the use of non-viral gene transfection due to viral-associated concerns, mostly regarding safety, such as induced inflammatory response and mutagenesis, as well as limitations on the plasmid size [4,5,6,7,8].

As gene therapy and stem cell therapy are closely related, very often using embryonic stem cells and stem cell lines for their clinical and research applications, MEFs have demonstrated a key role for the expansion and maintenance of embryonic stem cells as their feeder layers [9], as well as their different applications in tissue engineering [10,11]. Up to now, limited work has been done to investigate the potential of transfecting MEFs (NIH3T3 cells) for their use in tissue engineering and stem cell biology.

Here, we investigate the potential of the novel non-viral, nanomagnetic gene transfection in the presence of oscillating magnetic fields for NIH3T3 transfection.

Originally, magnetic nanoparticle-based gene transfection was demonstrated by Mah, Byrne *et al.* more than a decade ago [12,13,14,15]. The group used magnetic microspheres with GFP-carrying rAAV linked to the microspheres via heparin sulfate. The complex was magnetically targeted to a specific region of a culture of HeLa cells. The magnetic targeting enabled highly efficient uptake of the GFP gene into HeLa cells localized at the site of the applied magnetic field.

In further experiments, Plank, Scherer, Rosenecker and others developed magnetic nanoparticles for non-viral “magnetofection” use, in which plasmid DNA or siRNA is coupled directly to magnetic nanoparticles coated with charged polymers to which the plasmids adhere [14,15,16,17]. High-field, high-gradient permanent magnets placed beneath the culture plate rapidly draw the particle/DNA complex into contact with the cells in culture, where it is taken up via endocytosis.

In 2003, in order to improve the transfection efficiency of magnetofection-type transfection techniques, our group began developing a variation of magnetofection, which employs oscillating magnet arrays to promote more efficient transfection via mechanical stimulation of endocytosis. The technique generally improves transfection efficiency, but also maintains the advantages of magnetofection—rapid transfection and high cell viability in comparison to other non-viral transfection methods [18,19,20]. In addition, studies by our group and others on magnetic nanoparticles for regenerative medicine and gene transfection have shown that these techniques have little or no toxic effects on cells [19,20,21,22,23,24].

**Figure 1:**
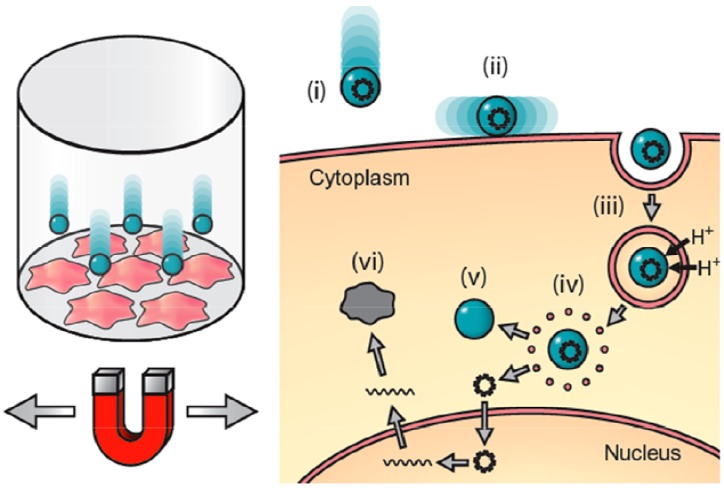
Principle of oscillating nanomagnetic transfection: Plasmid DNA or small interfering RNA (siRNA) is attached to magnetic nanoparticles and incubated with cells in culture (left). An oscillating magnet array below the surface of the cell culture plate pulls the particles into contact with the cell membrane (**i**) and drags the particles from side-to-side across the cells (**ii**); mechanically stimulating endocytosis (**iii**). Once the particle/DNA complex is endocytosed, proton sponge effects rupture the endosome (**iv**) releasing the DNA (**v**), which then transcribes the target protein (**vi**) [26,27].

The technique works by attaching DNA or siRNA to magnetic nanoparticles coated with a charged polymer, which condenses the DNA on the surface. The complex is placed into cell culture plates and above the oscillating high-field, high-gradient magnet array system (magnefect-nano™), which pulls the particle/DNA complex into contact with the cells. The added lateral motion promotes efficient uptake of the complex via endocytosis. Once internalized, the polymer acts as a proton sponge, rupturing the endosome due to osmotic pressure changes. This follows the release of the particles from the endosome and the DNA from the particles (Figure 1). The magnefect-nano™ system enhances transfection efficiency and protein expression [18,19,25], and in this study, we evaluated its potential as a method for non-viral NIH3T3 transfection.

## 2. Materials and Methods

### 2.1. Cell Culture

NIH3T3 cells were cultured in DMEM culture medium (w Hepes & Glucose, w/o L-Glutamine) (BioWhittaker, Fisher Scientific, UK), supplemented with 10% calf serum (Lonza, UK), 100 U/mL penicillin (Lonza, UK), 0.1 mg/mL streptomycin (Lonza, UK), 0.25 μg/mL phortericin B (Lonza, UK) and 2 mM L-glutamine (Lonza, UK). Before transfection, 1 × 10^4^ cells per well were seeded onto 96 well tissue culture plates and incubated at 37 °C and 5.0% CO_2_ for a period of 24 h to allow cells to adhere to the bottom of the wells.

### 2.2. Nanoparticle: DNA Optimization and Binding

The optimal MNP:DNA ratio for NIH3T3 transfections was obtained by investigating the capacity of nTMag MNPs (nanoTherics Ltd., Stoke-on-Trent, UK) to bind pEGFP-N1 plasmid endotoxic free DNA coding for GFP) (Plasmid Giga Kit, QiaGen, West Sussex, UK) in Knock-out DMEM media (Gibco-Invitrogen, UK) using spectrophotometry. nTMag MNPs are biocompatible and biodegradable, 100 nm in diameter, superparamagnetic nanoparticles (when used as per manufacturer’s instructions) with magnetite cores coated with proprietary multilayer PEI derivate. nTMag MNPs have been provided with accompanying quality control information that includes pH value of suspension (7.0), particle size distribution (1.7) and zetapotential (+23.87 mV). A range of different nTMag concentrations were mixed with a fixed volume of pEGFP-N1 plasmid (30 μL). The proportion of DNA that remained unbound (*i.e.*, still in solution after centrifugation) was then determined. A sample containing only DNA and Knock-out DMEM media, but no nTMag MNPs, was assayed as a control. Following mixing of the MNPs and DNA at room temperature (RT) for 15 min, samples were centrifuged at 14,000 rpm for 5 min to induce sedimentation of the particles and/or particle:DNA complexes. The absorbance of the supernatant containing the unbound DNA was measured for each sample at 260 nm and compared with the Knock-out DMEM blank sample. In order to determine the proportion of unbound (free) DNA, absorbance readings were expressed as percentages of the absorbance of the DNA-only (blank) control.

### 2.3. Transfection of NIH3T3 Cells Using the Magnefect-Nano System

Experimental evaluation of transfection efficiency was performed using transfection complexes composed of 100 μL of Knock-out DMEM medium, 0.2 μg DNA and 0.2 μL nTMag MNPs per 96 well tissue culture plate well (Sigma-Aldrich, Dorset, UK). Following the addition of the particle/DNA complexes to the cell cultures, samples were transferred to an incubator at 37 °C, 5% CO_2_ and placed over the magnefect-nano oscillating magnet array (nanoTherics Ltd., Stoke-on-Trent, UK) for 30 min. At 30 min post-transfection, the cell culture plates were removed from the magnefect-nano system, and transfection complexes were replaced with 100 μL of supplemented medium (as described previously). All samples were transferred back into an incubator for 48 h before analysis.

### 2.4. Transfection of NIH3T3 Cells with Lipofectamine 2000

NIH3T3 cells were maintained as described previously and seeded onto 96 well tissue culture plates. Lipofectamine 2000 complexes were composed of 100 μL Knock-out DMEM medium, 0.2 μg pEGFP-N1 DNA and 0.5 μL Lipofectamine 2000 per 96 well tissue culture plate well, as per the manufacturer's recommended protocol. Following the addition of complexes, samples were transferred into an incubator at 37 °C, 5% CO_2_, and complexes were left on the cell cultures for 6 h, as per the manufacturer’s protocol. At 6 h post transfection, all transfection complexes were replaced with 100 μL supplemented medium (as described previously). All samples were transferred back into an incubator for 48h before analysis.

### 2.5. Immunofluorescence & Fluorescence Activated Cell Sorting (FACS) Assays

Following 48 h of incubation, immunofluorescent microscopy was performed to acquire fluorescent images of NIH3T3 cells labeled with Phalloidin-TRICH actin stain (Sigma-Aldrich, Dorset, UK) and GFP from GFP-expressing transfected NIH3T3 cells, as shown in Figure 3. Images were captured with a Nikon Eclipse Ti fluorescent microscope at fixed exposure settings and magnification (20×) and processed with NIS-Elements software.

To quantitatively determine the transfection efficiency of nanomagnetic gene transfection and lipid-mediated gene delivery, 48 h after treatment NIH3T3 cells were washed with PBS, trypsinized and resuspended in PBS. FACS was performed on at least 50,000 cells per sample using a FACSort flow cytometer (Beckton Dickinson, Oxfordshire, UK) and data was analyzed using the CellQuest package (BD Biosciences).

### 2.6. Cell Viability

Following 48 h incubation post-transfection, samples were assayed using a Cytotox-ONE Homogeneous membrane integrity assay (Promega, Southampton, UK) for their cell viability, as per the manufacturer’s protocols. This method was used to assess membrane integrity of the cells as a proxy for viability following transfection by measuring LDH release from cells into the culture medium.

### 2.7. Statistical Analysis

Data from transfection efficiency and cell viability were analyzed using the GraphPad InStat 3 statistical analysis package. Statistical significance of the data was analyzed by a one-way ANOVA using a Tukey-Kramer multiple comparison test.

## 3. Results

### 3.1. DNA Binding to MNPs and Transfection of NIH3T3 Cells

**Figure 2:**
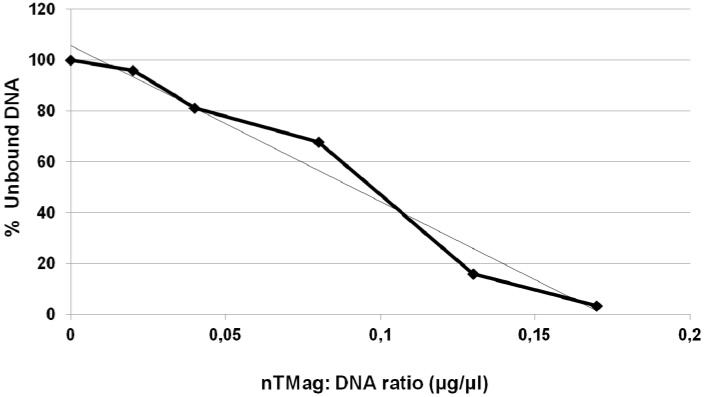
DNA binding curve showing the nTMag:pEGFP-N1 plasmid ratio of binding.

DNA binding assays demonstrated that nTMag MNPs efficiently bind pEGFP-N1 plasmid DNA. The nanoparticle: Plasmid DNA ratios used for the transfection experiments were those that reached particle saturation levels, and therefore, the particles had fully bound all the given concentration of plasmid DNA (Figure 2).

**Figure 3:**
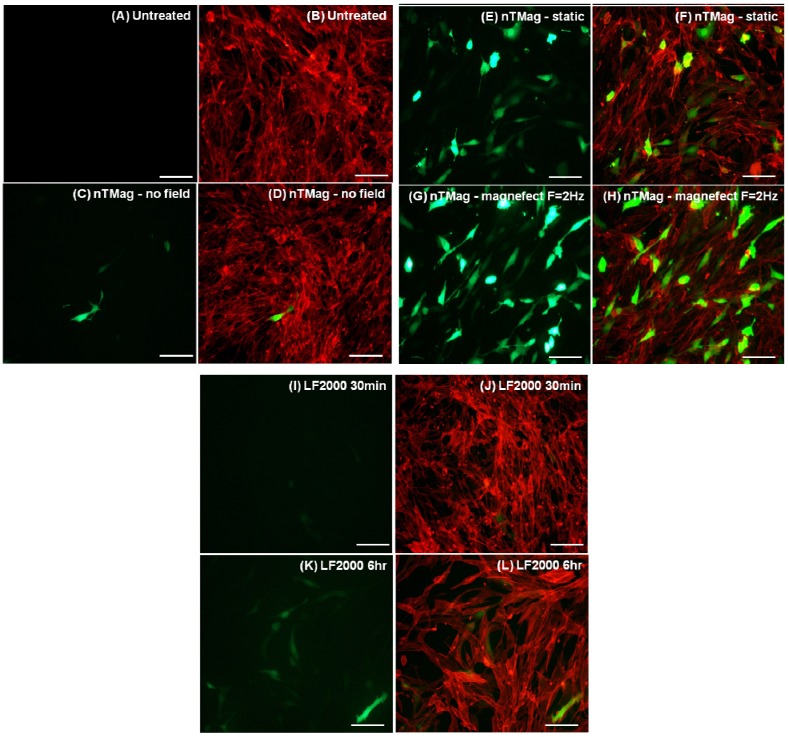
Fluorescent microscopy images of NIH3T3 cells expressing GFP and correspondingly labeled with Phalloidin for actin stain of the whole cell population. (**A**,**B**) Untreated (**C**–**L**) transfected with 100 nm nTMag MNPs coated with pEGFPN1 plasmid DNA; in the absence of a magnetic field, for 30 min (**C**,**D**), in the presence of a static field (nanoTherics static array) for 30 min (**E**,**F**) and an oscillating field (nanoTherics magnefect-nano™ array at *f* = 2 Hz and amplitude = 200 µm), for 30 min (**G**,**H**), Lipofectamine 2000™ for 30 min (**I**,**J**) and Lipofectamine 2000™ for 6 h (**K**,**L**). Cell seeding density was 1 × 10^4^/96 well, incubation period (48 h, 37 °C, 5% CO_2_) post-transfection and scale bar = 100 μm in (**A**–**L**). GFP: green fluorescent protein; nTMag MNPs: nanoTherics nTMag magnetic nanoparticles; F: oscillation frequency.

Fluorescence microscope images show a clear increase and similar GFP-expression levels between the samples transfected with nTMag MNPs exposed to magnetic fields, both static and oscillating (magnefect-nano™) for 30 min, in comparison to those transfected in the absence of a magnetic field and with Lipofectamine 2000 for 30 min and 6 h (Figure 3).

**Figure 4:**
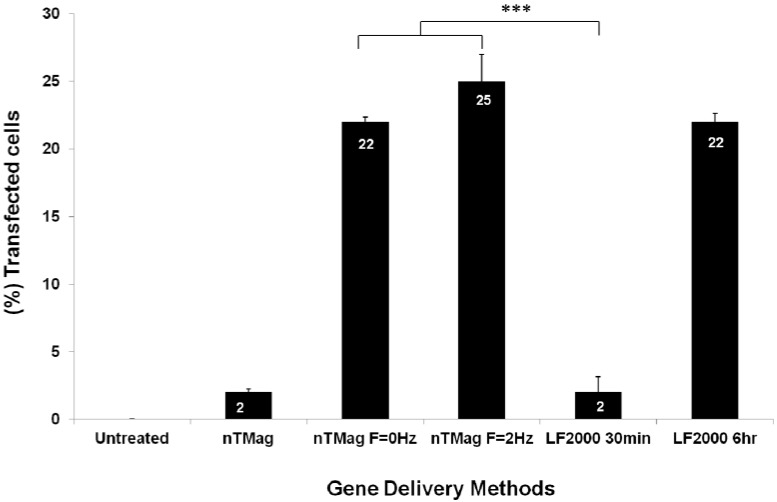
Average of FACS data from transfection efficiency of GFP-expressing NIH3T3 cells transfected with nTMag/GFP-DNA complexes in the absence of a magnetic field, in the presence of the nanoTherics static magnetic array and the nanoTherics magnefect-nano™ oscillating magnetic array (200 µm amplitude, *f* = 2 Hz) for 30 min, in comparison to lipid-based transfection (LF2000, for 30 min and 6 h). During transfection, cells were incubated at 37 °C and 5% CO_2_. At 30 min post-transfection, the magnets were removed, and cells were incubated for 48 h before analysis. *n* = 6 for all samples.FACS: Fluorescence activated cell sorting; GFP: Green fluorescent protein; F: oscillation frequency.

Quantitative analysis of transfection efficiency obtained using Fluorescence-Activated Cell Sorting (FACS) provided analogous results to those obtained via fluorescence microscopy. In the bar chart, the effect of the oscillating magnet array (*f* = 2 Hz) using nTMag MNPs at 30 min was compared to “no field” and “static field” (*f* = 0) conditions (at 30 min), as well as lipid-mediated gene transfection method at 30 min and 6 h (Figure 4).

Some GFP expression (2% TE) was observed in the presence of nTMag/GFP-DNA complexes even in the absence of a magnetic field. However, a clear increase in GFP-expressing NIH3T3 cells (25% TE) was demonstrated when nTMag/GFP-DNA complexes were introduced to the oscillating magnetic field (magnefect-nano system, nanoTherics) and to a static magnetic field (22% TE). The increase in transfection efficiency observed between the no field, and both the static and oscillating field conditions was found to be statistically significant (***p < 0.001). Furthermore, the use of both the static and oscillating field conditions significantly increased TE in comparison to Lipofectamine 2000 at 30 min (***p < 0.001), but was found to have analogous results to Lipofectamine 2000 at 6 h (22% TE), highlighting further the efficiency of the magnetic field systems at shorter transfection times (Figure 4).

### 3.2. Evaluation of MNPs Toxicity and Determination of Transfected NIH3T3 Cells Viability

**Figure 5:**
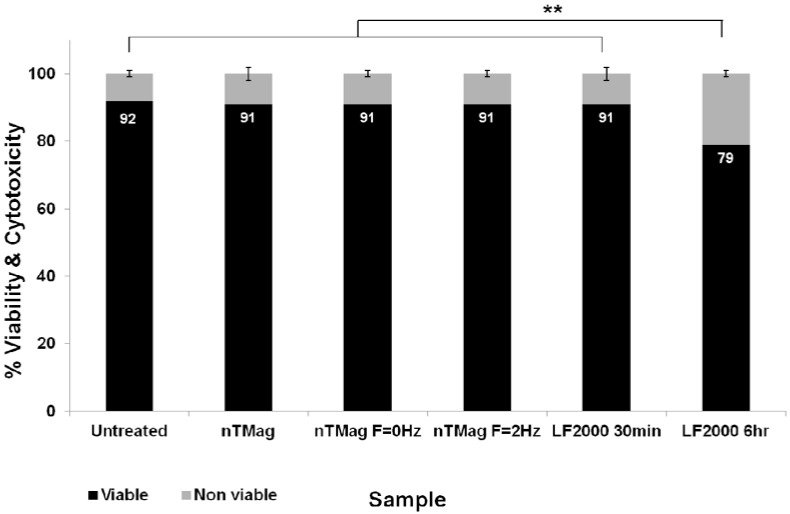
Bar chart showing combined average percentage for viable and non-viable cells following treatment with nTMag MNPs and “no field”, “static field” (*f* = 0 Hz) and “magnefect-nano” (*f* = 2 Hz) transfections at 30 min, compared with Lipofectamine 2000 at 30 min and 6 h, at 48 h post-transfection. *n* = 9 for all groups.

At 48 h post-transfection, the cell viability and the corresponding toxicity of the MNPs for no field, static and oscillating field conditions were tested using a Cytotox-ONE Homogeneous membrane integrity assay that measures LDH release from cells with non-intact cell membranes. These results were compared with the equivalent data for Lipofectamine 2000 at 30 min and 6 h, using the same cell viability assay. From the cell viability/toxicity bar chart, it was demonstrated that cell viability of transfected NIH3T3 cells using nTMag MNPs at no field, static and oscillating magnetic field conditions at 30 min were very similar to the untreated controls retaining high cell viability (Figure 5).

Transfected NIH3T3 cells with Lipofectamine 2000 at 30 min were found analogous with all the MNP-treated and untreated samples, however, significantly lower cell viability/higher toxicity was obtained when comparing these samples with transfected NIH3T3 cells with Lipofectamine 2000 for 6 h (**p < 0.01).

## 4. Discussion and Conclusions

The data presented here show that nanomagnetic transfection provides a fast and efficient method for non-viral transfection of mouse embryonic fibroblasts (NIH3T3). The method achieves in 30 min higher transfection efficiency than the 6 h treatment with the lipid-based reagents, retaining excellent cell viability levels comparable to untreated samples and significantly higher cell viability in comparison to Lipofectamine 2000 at 6 h.

Nanomagnetic gene transfection using mechanically stimulated particle/DNA uptake can be further improved by systematically investigating a wider range of frequencies and amplitudes. The method has great potential for use as an effective, non-viral transfection agent for MEFS. A major advantage of this technique for use in regenerative medicine and tissue engineering research applications is the fact that it is both non-viral and does not impact cell viability, though a thorough investigation of up- or down-regulation of off-target genes will be needed before the technique could be used in a clinical setting.
